# A Portable Real-Time Ringdown Breath Acetone Analyzer: Toward Potential Diabetic Screening and Management

**DOI:** 10.3390/s16081199

**Published:** 2016-07-30

**Authors:** Chenyu Jiang, Meixiu Sun, Zhennan Wang, Zhuying Chen, Xiaomeng Zhao, Yuan Yuan, Yingxin Li, Chuji Wang

**Affiliations:** 1 Institute of Biomedical Engineering, Chinese Academy of Medical Sciences & Peking Union Medical College, Tianjin 300192, China; jcy262626@163.com (C.J.); meixiu_sun@126.com (M.S.); zwang535@gmail.com (Z.W.); zhuying_tij@126.com (Z.C.); zhaoxiaomeng11@126.com (X.Z.); crds-bme@126.com (Y.Y.); 2 Department of Physics and Astronomy, Mississippi State University, Starkville, MS 39759, USA

**Keywords:** cavity ringdown breath analyzer, breath acetone, high data throughout, GC-MS validation, elevated mean acetone concentration

## Abstract

Breath analysis has been considered a suitable tool to evaluate diseases of the respiratory system and those that involve metabolic changes, such as diabetes. Breath acetone has long been known as a biomarker for diabetes. However, the results from published data by far have been inconclusive regarding whether breath acetone is a reliable index of diabetic screening. Large variations exist among the results of different studies because there has been no “best-practice method” for breath-acetone measurements as a result of technical problems of sampling and analysis. In this mini-review, we update the current status of our development of a laser-based breath acetone analyzer toward real-time, one-line diabetic screening and a point-of-care instrument for diabetic management. An integrated standalone breath acetone analyzer based on the cavity ringdown spectroscopy technique has been developed. The instrument was validated by using the certificated gas chromatography-mass spectrometry. The linear fittings suggest that the obtained acetone concentrations via both methods are consistent. Breath samples from each individual subject under various conditions in total, 1257 breath samples were taken from 22 Type 1 diabetic (T1D) patients, 312 Type 2 diabetic (T2D) patients, which is one of the largest numbers of T2D subjects ever used in a single study, and 52 non-diabetic healthy subjects. Simultaneous blood glucose (BG) levels were also tested using a standard diabetic management BG meter. The mean breath acetone concentrations were determined to be 4.9 ± 16 ppm (22 T1D), and 1.5 ± 1.3 ppm (312 T2D), which are about 4.5 and 1.4 times of the one in the 42 non-diabetic healthy subjects, 1.1 ± 0.5 ppm, respectively. A preliminary quantitative correlation (R = 0.56, *p* < 0.05) between the mean individual breath acetone concentration and the mean individual BG levels does exist in 20 T1D subjects with no ketoacidosis. No direct correlation is observed in T1D subjects, T2D subjects, and healthy subjects. The results from a relatively large number of subjects tested indicate that an elevated mean breath acetone concentration exists in diabetic patients in general. Although many physiological parameters affect breath acetone, under a specifically controlled condition fast (<1 min) and portable breath acetone measurement can be used for screening abnormal metabolic status including diabetes, for point-of-care monitoring status of ketone bodies which have the signature smell of breath acetone, and for breath acetone related clinical studies requiring a large number of tests.

## 1. Introduction

Breath analysis by testing the volatile organic compounds (VOCs) of exhaled breath, one of the three key clinical diagnostic techniques that have been used in Eastern Medicine for more than three thousand years, provides a potential non-invasive method for disease diagnosis, therapeutic monitoring, and metabolic status monitoring [1,2,3,4,5]. To date, there are more than 2000 VOCs in low concentrations, from parts per million (ppm) to parts per billion (ppb) or parts per trillion (ppt), identified to be present in exhaled human breath. Breath acetone has long been known as a breath biomarker for diabetes [6,7,8,9,10,11,12,13,14,15,16,17]. Effort on breath acetone analysis using various methods and technologies toward diabetes diagnostics and monitoring has been made over the past 50 years. However, huge variations exist among the results from different studies [6]. One of the main reasons is that those studies used a limited number of human subjects or samples, which is partially due to high costs of breath analysis using the convention analytical methods such as GC-MS and long testing times resulting from sophisticated sample preparation such as sample pre-concentration as required by the methods. To this end, it is highly desirable to have a high data throughput instrument or method that is able to test a sufficiently large number of diabetic subjects in a relatively short experimental time. 

Recent efforts in pursuits of a real-time on-line breath analyzer with high data throughput are fruitful [9,11,18,19,20,21,22,23,24,25,26,27,28]. Several relatively new MS-based analytical techniques have been developed and tested for on-line breath analysis, including proton transfer reaction mass spectrometry (PTR-MS) [22,23], vacuum-free ion mobility spectroscopy (IMS) [24,25], and selected ion flow tube mass spectrometry (SIFT-MS) [9,11,26]. Breath analysis is also conducted by using electrical sensors, which are comparatively inexpensive and smaller in size [27,28], but the issue with detection selectivity and requirement of frequent baseline calibration remain to be further addressed.

More recently, to pursue a large scale of clinical testing in near-real time, on-line toward high data throughput, we have designed and constructed a breath acetone analyzer based on the cavity ringdown spectroscopy (CRDS) technique [29,30,31,32,33,34,35]. As a laser spectroscopy-based technique, CRDS has been successfully used for trace gas analysis including breath analysis. Compared to the use of the GC-MS method, the CRDS method is well accepted for trace gas analysis on account of its advantages of high sensitivity, high accuracy, real-time response without need of sample pre-process, and relatively low cost, which make the technique both scientifically and economically attractive for breath analysis, particularly when a large number of subjects have to be tested. Now a ringdown breath acetone analyzer has been developed recently and applied to measure breath acetone concentration of human subjects in a laboratory and a clinic. Breath acetone in human, canine, and muridae subjects has been measured using the CRDS technique in the previous studies [35,36,37,38,39,40,41]; however, none of the large volume of data (1000 or more) was measured by a real-time on-line ringdown breath acetone analyzer.

In this work, we update the status of the development of a standalone ringdown breath acetone analyzer and its clinical application in measurements of breath acetone from 22 hospitalized Type 1 diabetic (T1D) patients, 312 hospitalized Type 2 diabetic (T2D) patients, and 52 non-diabetic healthy subjects under various situations (fasting or post-meals). Thousands of data collected by the real-time on-line standalone ringdown breath analyzer in a short experimental period are reported here. Potential applications of the real-time portable breath acetone analyzer (LaserBreath-001) in diabetes screening and management are discussed.

## 2. Experimental

### 2.1. Ringdown Breath Acetone Analyzer

**Figure 1:**
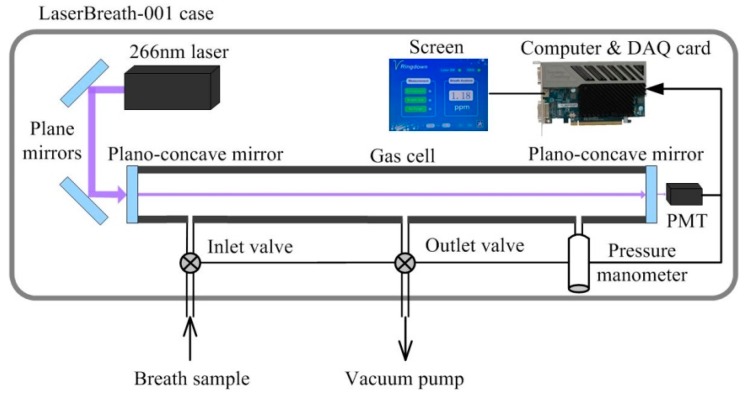
Schematic of the integrated standalone CRDS breath acetone analyzer (LaserBreath-001).

**Figure 2:**
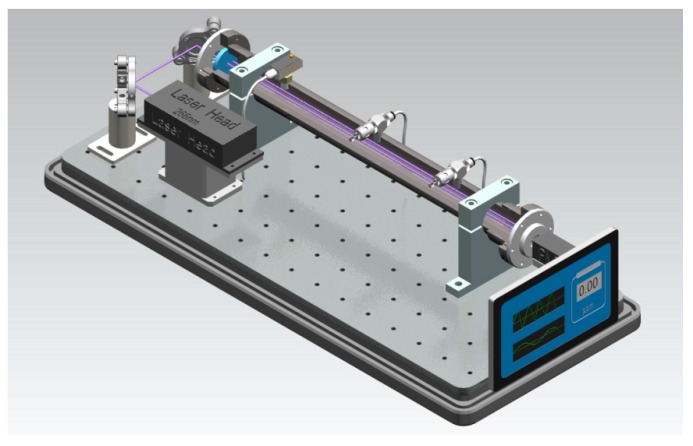
Schematic diagram of the optical layout in the ringdown breath analyzer.

The integrated standalone breath acetone analyzer (LaserBreath-001) developed in our lab is illustrated in Figure 1. All displays, including the ringdown decay waveform and real-time ringdown signals, are controlled via the touch screen. Figure 2 shows a schematic diagram of the optical layout of in the breath acetone analyzer. A Q-switch Nd:YAG laser (Changchun New Industries Optoelectronics, China) equipped with a compact laser head (21.0 mm × 88 mm × 74 mm) was employed as the light source in the breath analyzer. The laser operated at 266 nm with a repetition rate of 1 kHz and single-pulse energy of 4.5 µJ. Except for an iris and two reflective mirrors (Thorlabs PF05-03-F01, Newton, NJ, USA) used to direct the laser beam, the output of the laser was coupled into the ringdown cavity without using any mode-matching optical parts. The gas cell, was comprised of a 50-cm-long stainless steel pipe with an inner diameter of 3.81 cm (CRD Optics Inc., Lompoc, CA, USA), one pair of mirror mounts (CRD Optics Inc., Lompoc, CA, USA), and one pair of high-reflectivity UV mirrors (Los Gatos Research, San Jose, CA, USA, R = 99.99%, radius of curvature = 1 m). Each mirror mount held a mirror whose position could be adjusted in multiple dimensions via three alignment screws. As shown in Figure 2, three ports were located at this gas cell, one was used as a gas inlet and another two were gas outlets, which were connected to a vacuum pump (Oerlikon, SC5D, Cologne, Germany) and a micro pressure manometer (MKS, 870B, Andover, MA, USA) used to monitor the inside cell pressure. In order to ensure the gas cell protected from contaminated, high-purity nitrogen (>99.99%, DaFang Special Gas, Tianjin, China) was used to flush the gas cell each time after one breath sample was tested. A miniature photomultiplier tube (PMT) (R74000U-09, Hamamatsu, Japan) was used as the detector to capture the ringdown decay waveform that was input to an oscilloscope (Tektronix TDS3012B, Beaverton, OR, USA) for display.

A ringdown waveform was digitalized to 10,000 data points and transferred to the in-house electronic portion of the instrument. The in-house developed ringdown software was used to obtain a ringdown time by fitting the ringdown data points to a single exponential decay.

### 2.2. Measuring Method

The background subtraction method, described in our previous works [39,40,41,42,43], was applied to obtain breath acetone concentrations. In this method, the optical cavity loss caused by the laboratory atmosphere at 1 atm is defined as the effective absorbance of the atmosphere, as described by the equation:

(1) $$A_{\text{atm}}=\sigma_{266}nd=\frac{d}{c}\left(\frac{1}{\tau_{\text{atm}}}-\frac{1}{\tau_{0}}\right)$$

where σ_266_ is the absorption cross-section of acetone at 266 nm, i.e., 4.5 × 10^−20^ cm^2^/molecules at atmospheric pressure and room temperature, *n* is the acetone concentration, *d* is the distance between the two mirrors, *c* is the speed of light in a vacuum, τ_atm_ and τ_0_ are the ringdown times when the gas cell is filled with laboratory air to 1 atm and under vacuum, respectively. Likewise, the absorbance of breath acetone is obtained by

(2) $$A_{\text{breath}}=\sigma_{266}nd=\frac{d}{c}\left(\frac{1}{\tau_{\text{breath}}}-\frac{1}{\tau_{0}}\right)$$

where *A*_breath_ is the absorbance of the breath gas, τ_breath_ is the ringdown time detected when the gas cell is filled with breath gas.

In this study, by using the laboratory atmosphere as the background, the absolute concentration of breath acetone (the upper limit) in the breath gas is obtained by

(3) $$\Delta A=A_{\text{breath}}-A_{\text{atm}}=\sigma \left(\nu \right)nd$$

where Δ*A* is the absorbance difference.

It should be noted that the breath acetone concentrations obtained by the background subtraction method are the upper limits of acetone concentration in the breath samples. The rationale of the background subtraction method includes an assumption that the absorbance difference is attributed to the absorption of acetone alone. The reliability of this assumption has been rigorously evaluated in a previous study by investigating the possible absorbance from other atmospheric molecules and high abundance breath volatile organic compounds (VOCs) including several atmospheric molecules (H_2_O, N_2_, O_2_, and CO_2_), high concentration breath gases from 0 to 10 ppm (NH_3_, CH_4_, NO, N_2_O and CO), and high abundance breath VOCs (C_5_H_8_, CH_3_OH, C_2_H_5_OH, C_3_H_8_O, C_2_H_6_ and C_5_H_12_). The contribution of the highest abundance VOC, isoprene (C_5_H_8_), to the absorption at 266 nm is about 200 times smaller than that of acetone in normal human breath [37,38,42]. Other breath VOCs mentioned above have no or negligible absorption compared with acetone at 266 nm.

### 2.3. Breath Analysis Volunteers and Patients

In this study, all healthy participants were volunteers. T1D and T2D subjects were hospitalized patients in the Metabolic Diseases Hospital, Tianjin Medical University, China. The research procedures and activities followed the research protocols approved by the Institutional Review Board (IRB approval number: IRB2013-053-01) of human subject research in Tianjin. All volunteers and patients agreed to perform the experiments (signed an informed consent form). The more detailed information including process was shown in the supplementary material [41].

A subject took a deep breath and breathed a single breath via a disposable mouthpiece into a fluorinated ethylene propylene (FEP) breath-gas-collection bag (~1 L), which was cleaned using high-purity nitrogen (>99.99%) prior to use. Simultaneous BG levels were measured using a standard diabetic-management blood glucometer (Roche, Accu-Chek Performa, Basel, Switzerland). The breath sample collected in the FEP breath bag was introduced into the gas cell through a section of quarter-inch tubing connected to the gas inlet of the analyzer. It was tested that the FEP bag can keep breath gas fresh for up to 6 h. No additional procedure was used to handle excessive moisture in the exhaled breath since water molecules have no absorption at 266 nm.

## 3. Results and Discussion

### 3.1. Performance of the Instrument

**Figure 3:**
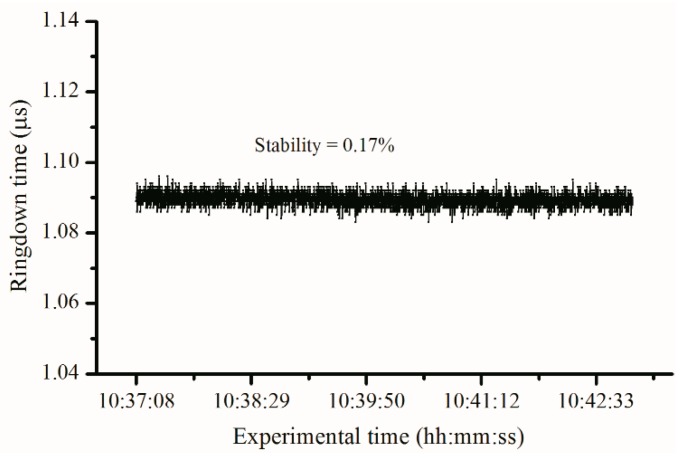
The typical instrument stability in terms of the ringdown time obtained. Each data point is generated from averaging 100 ringdown events, a stability σ/$\bar{\tau }$ of 0.17% was measured.

**Figure 4:**
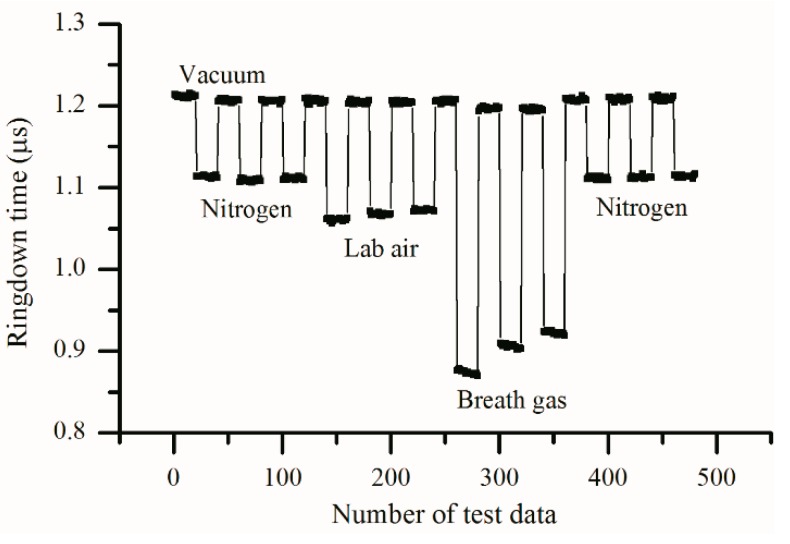
The instrument shows good reproducibility in terms of the ringdown times obtained at different pressures: 0 Torr, 730 Torr nitrogen, and the laboratory atmosphere.

After the breath analyzer was constructed, tests were conducted to check its properties including stability, reproducibility, response time, and the limit of detection. The ringdown baseline scans were obtained in order to test the instrument’s stability. The instrument stability is defined as σ/$\bar{\tau }$ where σ and $\bar{\tau }$ are the standard deviation and average of ringdown time, respectively. The internal pressures of the gas cell were set at low and high levels, i.e., 0 Torr and 730 Torr, by introducing different quantities of laboratory air into the gas cell. As shown in Figure 3, a typical baseline scan of the LaserBreath-001 was obtained with each data point to be generated from an average over 100 ringdown events. The typical baseline stability is better than 0.5%. The mirror reflectivity was characterized to be 99.9% by measuring ringdown time in an empty cell. Figure 4 shows the analyzer’s response to laboratory air at 730 Torr. The sharp rising and falling edges of the signals indicate the rapid response of the analyzer. This test demonstrates the good reproducibility and fast response of the breath acetone analyzer. The decrease in ringdown time when nitrogen and laboratory air fills a vacuumed cavity is due to the Rayleigh scattering at 266 nm, this Rayleigh scattering loss is self-corrected using the background subtraction method [37]. Given the known absorption cross-section of acetone at atmospheric pressure, σ_266nm_ = 4.5 × 10^−20^ cm^2^/molecule, using the ringdown baseline stability, 0.5%, and the mirror reflectivity, 99.99%, the theoretical detection limit of the analyzer for acetone is determined to be 30 ppb (parts per billion) based on the 3 − σ criterion.

**Figure 5:**
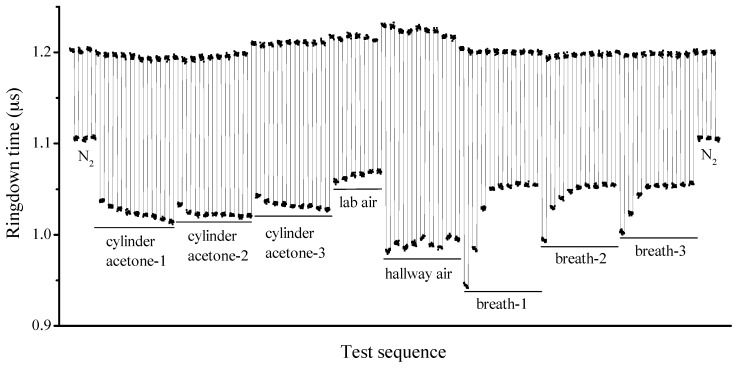
The instrument response to various samples, such as cylinder nitrogen, cylinder acetone helium mixture, laboratory air, hallway air, and breath samples.

It should be noted that the breath acetone analyzer gave consistent results in the vacuum conditions, measurements of nitrogen, and the laboratory air. However, the successive measurements of a breath samples showed a consistent decrease in acetone concentrations (increase in obtained ringdown times) (in Figure 4). The instability came primarily from the humidity variation of the breath sample. Due to the humidity loss of a breath sample after it was collected and stored in a breath bag, the obtained ringdown times increased in the three successive measurements, and this phenomenon was more obvious when the breath sample was tested right after collection, as can be seen in Figure 5. Three bags of breath sample were collected from the same subject and tested immediately, which were marked as breath-1, breath-2, and breath-3 in Figure 5. The first measured results of these three samples were all dramatically higher (lower in terms of ringdown times) than those that followed, and then reached stable values gradually. In order to confirm the effect of humidity on measured results, cylinder nitrogen, cylinder acetone samples (acetone helium mixture), and atmospheric air were tested. Cylinder nitrogen, laboratory air, and hallway air were tested using the instrument directly, while the cylinder acetone samples were transferred into three bags and tested immediately. The obtained results show that measured ringdown times of nitrogen and acetone samples were consistent, respectively. However, the ringdown times of laboratory air were longer than those of hallway air because the laboratory humidity is lower due to the air cooling and the dehumidification system. Further note that the effect of humidity on the acetone measurements is not due to absorption of water, instead it is due partially to the influence of the breath moisture on the reflectivity of the ringdown mirrors.

**Figure 6:**
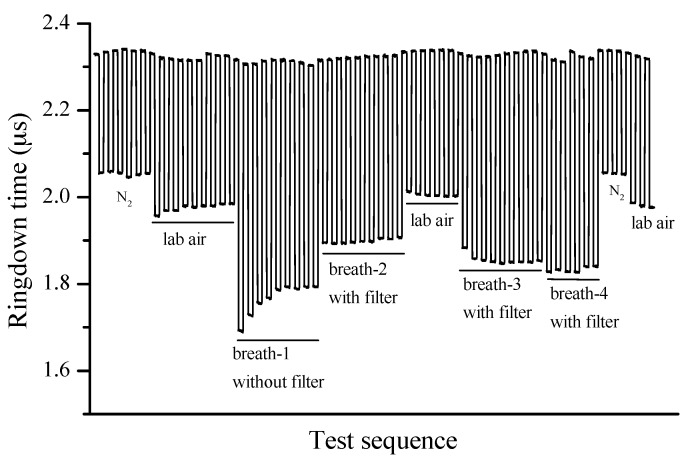
The instrument response to various samples and the effectiveness of humidity removal using a membrane filter.

For the purpose of reducing high humidity in the breath samples, a membrane filter with a pore size of 0.22 μm was connected to the instrument inlet port. Figure 6 shows its humidity removal effectiveness. Four breath samples were collected from the same subject and tested right after the breath gas collection. The same humidity effect was observed when a breath sample (marked as breath-1) was tested without using a membrane filter, but the results of another three samples (marked as breath-2, breath-3, and breath-4) were different from the breath-1 sample yet consistent among the three since a membrane filter was used.

### 3.2. Comparison of Acetone Values from CRDS to GC-MS Values

In order to investigate the accuracy of the analyzer, six breath samples from four individual healthy subjects and five breath samples from three T2D subjects were tested using both the ringdown breath acetone analyzer and a certified GC-MS facility. In this experiment, breath samples including fasting (14-h overnight fast), after breakfast, after lunch, and after dinner from the healthy and the T2D patients were taken. The GC-MS analysis was conducted in the State Key Laboratory on Odor Pollution Control, Tianjin Academy of Environmental Science (TAES), which is a certified facility for trace chemical species analysis. As the Compendium Method TO-15, a cryogenic pre-concentrator (ENTECH Instruments, Inc., 7100A, Simi Valley, CA, USA) was employed to remove N_2_, CO_2_, and H_2_O.

The VOCs-enriched sample was introduced into the GC-MS system operated in the selected ion monitoring (SIM) mode with a higher sensitivity and better signal-noise ratio. The operating conditions and analytical conditions of the GC-MS system can be seen elsewhere. It took about one hour to test one sample. Considering high testing costs including the long testing time, the measured acetone concentrations using the methods (the analyzer and the GC-MS) were only up to 2.5 ppm that covers the major range of acetone concentration in healthy and T2D subjects.

**Figure 7:**
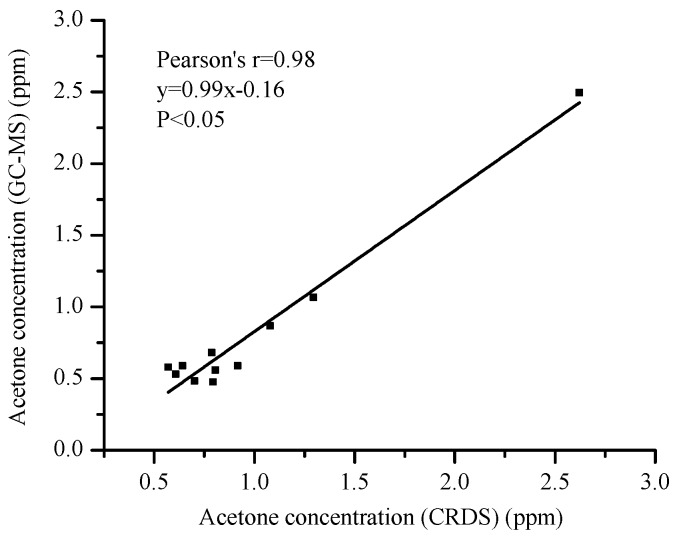
The ringdown breath analyzer’s performance validated by a certified GC-MS facility.

Figure 7 shows the results and they agree with each other, with a linear fitting coefficient of 0.98. The slope (0.99) suggests that the obtained acetone concentrations via both methods are consistent. This GC-MS validation test proves that the ringdown breath acetone analyzer is reliable and fast, which can be used for subsequent measurements of breath samples.

### 3.3. Breath Tests in Human Subjects Using the Validated Breath Acetone Analyzer

52 non-diabetic healthy subjects, 22 T1D, and 312 T2D patients participated in this study over a period of two months. Table 1 lists the detailed information on the 1257 breath samples collected from the 386 subjects, for example, in the 22 T1D subjects, the number of the subjects who provided 1, 2, 3, and 4 breath samples is 2, 2, 2, and 16, respectively. In total, 76 breath samples were collected from the T1D subjects. Table 2 lists the statistical information and test results (n1, n2, and n3 designate T1D, T2D, and healthy subjects, respectively). It should be pointed out that the subtraction approach is feasible only if inspired/room air concentrations compared to exhaled concentrations are low (<5%). As a matter of fact, acetone concentrations in the atmosphere air ranged from 0 to 40 ppb in accordance with Toxicological Profile for acetone (U.S. Department of Health and Human Services, Agency for Toxic Substances and Disease Registry, 1994), which depends on the local air quality. The concentration is about 0.6%, 3%, and 2% of the mean breath acetone concentrations in T1D subjects (4.9 ± 16 ppm), T2D subjects (1.5 ± 1.3 ppm), and healthy subjects shown in Table 2, correspondingly.

**Figure 8:**
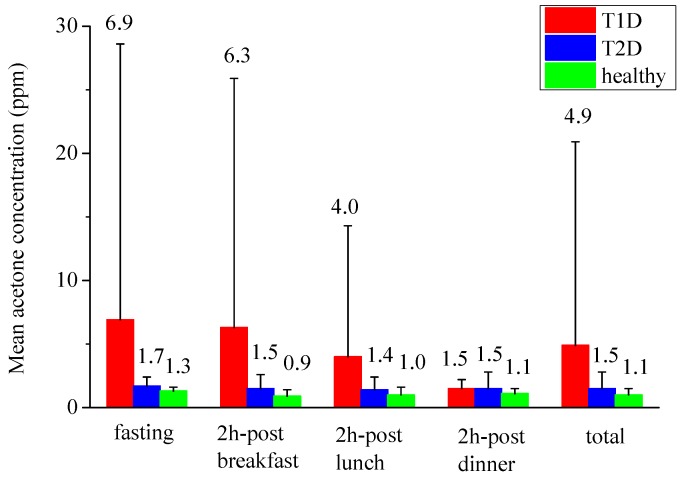
Mean breath acetone concentrations in 22 T1D subjects, 312 T2D subjects, and 52 non-diabetic subjects, under four different conditions: fasting, 2 h post-breakfast, 2 h post-lunch, and 2 h post-dinner. The error bar corresponds to one standard deviation.

In this study, up to a maximum of four types of breath samples from each individual subject (average 3.2 per subject) was collected and tested under four different conditions: fasting, 2 h-breakfast, 2 h post-lunch, and 2 h post-dinner. Figure 8 shows the mean breath acetone concentrations for the non-diabetic healthy subjects and diabetic subjects (T1D and T2D) under both fasting and 2 h post-meals. The breath acetone concentrations of the 52 non-diabetic subjects ranged from 0.1 to 2.0 ppm and the global mean over the 168 samples collected from the 52 healthy subjects was 1.1 ± 0.5 ppm. As shown in Table 2 and Figure 8, the ranges of breath acetone concentrations for the non-diabetic healthy subjects under the four different conditions were 0.3–1.9, 0.1–2.0, 0.1–2.0, and 0.3–1.4 ppm, respectively. The mean breath acetone concentrations for the healthy subjects under the four different conditions were determined to be 1.3 ± 0.3, 0.9 ± 0.5, 1.0 ± 0.6, and 1.1 ± 0.4 ppm, respectively. In general, the mean breath acetone concentration in the healthy subjects studied in this work is consistent with the published results, which have a range from 0.39 to 1.03 ppm [6]. It is normal that breath acetone concentrations in a few samples were 2.0 ppm. In fact, breath acetone concentrations can be affected on hourly and daily time scales by some factors including diet. After initiating a calorie restriction diet, breath acetone concentrations rise for 3–8 days (in subjects who were losing fat) before reaching a new steady state. In this work, all healthy subjects were not under the condition of the same diet. Whether the cause of the few higher breath acetone concentrations is due to the different diets or not remains to be studied.

The breath acetone concentrations of the 22 T1D and 312 T2D subjects involved in this work are shown in Figure 8, it ranged from 0.3 to 103.7 ppm, and 0.1 to 19.8 ppm, respectively. The mean values are 4.9 ± 16 ppm (T1D), and 1.5 ± 1.3 ppm (T2D), which are about 4.5 and 1.4 times of the mean value of acetone concentration in the 52 non-diabetic healthy human subjects, respectively. Four different ranges of breath acetone concentrations for the T1D subjects under the four different conditions are 0.7–103.7, 0.7–89.5, 0.3–42.5, and 0.6–2.9 ppm, respectively. The mean breath acetone concentrations under the four different conditions for the 22 T1D subjects are 6.9 ± 21.7, 6.3 ± 19.6, 4.0 ± 10.3, and 1.5 ± 0.7 ppm. Similarly, the four ranges of breath acetone concentrations for the 312 T2D are 0.1–19.8, 0.1–7.1, 0.1–7.2, and 0.1–10.6 ppm, respectively; and the four mean breath acetone concentrations for the T2D are 1.7 ± 0.7, 1.5 ± 1.1, 1.4 ± 1.0, and 1.5 ± 1.3 ppm, correspondingly. For the T1D and T2D subjects studied in this work, the mean values of all diabetic subjects under fasting or 2 h post-meals are all higher than that in the non-diabetic healthy human subjects, respectively. The result suggests that there is an elevation of mean breath acetone concentration for diabetic subjects no matter which of the four conditions they are under.

**Figure 9:**
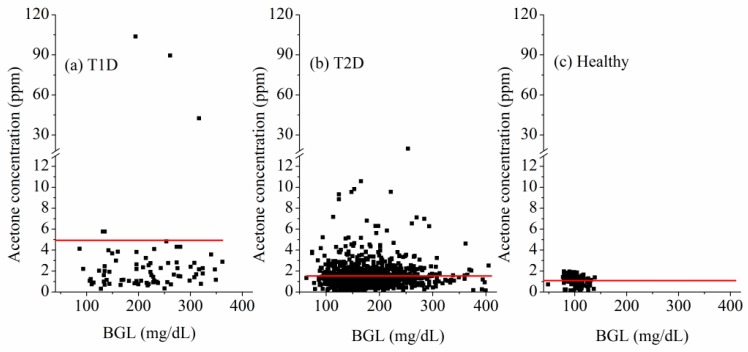
The measured breath acetone concentrations versus BG levels, (**a**) 22 T1D subjects; (**b**) 312 T2D subjects; and (**c**) 52 healthy subjects.

**Figure 10:**
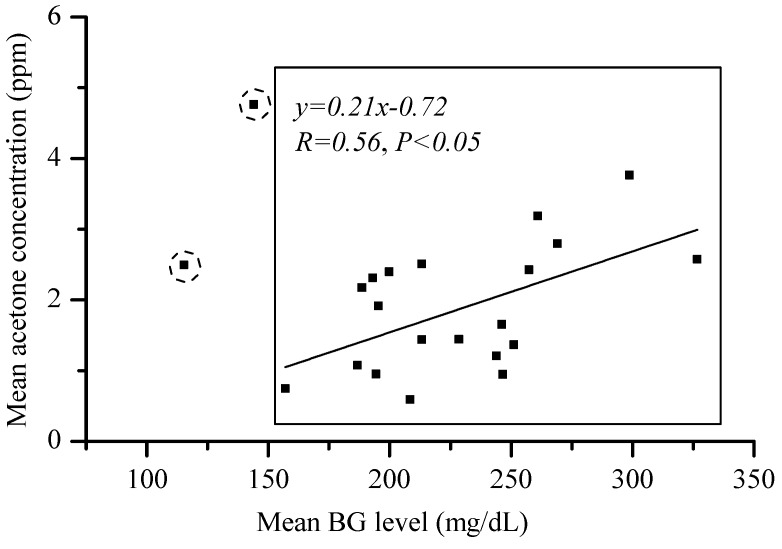
Observed correlation of the mean breath acetone concentration (ppm) with the mean blood glucose level in all T1D subjects with no ketoacidosis. The two samples with ketoacidosis are marked in dashed circles.

Figure 9a–c respectively show the breath acetone concentration versus the concurrent BG level of the 22 T1D subjects, 312 T2D subjects, and 52 non-diabetic healthy subjects involved in this work. No direct correlation between the measured individual breath acetone concentrations and the individual BG levels exists in the 22 T1D subjects, 312 T2D subjects, and 52 non-diabetic healthy subjects studied in this work, as shown in Figure 9a–c, correspondingly. However, for the 20 T1D subjects, all T1D subjects excluding two subjects with ketoacidosis, a linear correlation between the mean breath acetone concentrations and the mean BG levels was observed. A linear fitting yielded R = 0.56 and *p* < 0.05, as shown in Figure 10. The results are consistent with the results reported previously in the literatures based on the group methods [17,35]. The cause of some individuals deviating from the general trend needs to be further investigated.

In fact, neither a single study nor the collective results from over the last 50 years of study can give a conclusive answer to whether there is a quantitative relation between breath acetone and BG or not to date, even though grouped breath acetone concentrations based on the BG level and HbA1c showed some critical meaning in previous studies. The number of T1D or T2D subject accounts for only about 1% of the more than 3000 human subjects recruited collectively for quantitative breath acetone measurements based on the published literature [6]. Consequently, the number of diabetic subjects used in breath analysis in the literature is still obviously insufficient for the study of breath acetone concentrations in diabetic people and its correlation with blood glucose level. On top of that, there are huge variations among the results from different studies on account of no “gold-standard method” for breath acetone measurements due to technical problems of sampling and analysis. With the remarkable progress of analytical techniques and sampling methodology, a real-time on-line ringdown breath analyzer, the LaserBreath-001, a validated breath analyzer, was applied for measuring 1257 breath acetone samples from 52 non-diabetic healthy subjects and 334 diabetic patients (22 T1D and 312 T2D), which represented a relatively large number of diabetic subjects used ever in a single study. The results suggest that elevated mean breath acetone concentrations exist in diabetic subjects. In 20 T1D subjects with no ketoacidosis, a linear correlation between the mean individual breath acetone concentration and the mean individual BG levels is observed. No direct (without grouping and considering the group mean) correlation was found for between the breath acetone and the BG level for T1D subjects, T2D subjects, and healthy subjects in this work. As can be seen in Table 2, there are significant differences in the weight and body mass index (BMI) among the T1D group, the T2D group, and the non-diabetic healthy group. Both parameters were found to correlate to the breath acetone concentration in the previous studies. Comparatively, the relationship between a breath acetone level and a BG level and other bioinformatic parameters remains to be further investigated with a large number of subjects (e.g., 1000 s more). This type of breath analyzer, which will be further developed into an ideal tool for breath acetone measurement, is the key to obtaining a large pool of clinical data on breath acetone.

### 3.4. Toward Diabetes Screening and Management

Currently, diabetes screening still relies on the use of a pharmacy-based blood glucose meter (BGM). Readings from a commercial BGM have a large standard deviation and typically a BGM has an error bar up to 20% or larger when it is used for BG measurements, depending on how the testing steps are followed. Accordingly, the observed deviation in the BG levels of both healthy and diabetic subjects could be partially attributed to the measurement uncertainty of the used diabetic-management blood glucometer. Blood A1C test is therefore recommended as a further confirmation of the BGM results. In addition to the pain and inconvenience from the required blood sampling when using a BGM, collective costs of using a BGM cannot be underestimated, approximately one USD per measurement [44]. For diabetes management, daily frequent check of BG levels would cost much more. A low cost and noninvasive (no blood draw) diabetes screening and or diabetic status monitoring is highly desirable, yet such as device is still not available. Issues in using the breath acetone analyzer include: (1) Many physiological parameters and conditions affect breath acetone, such as fasting, excise, diets, exposure to high acetone industrial environments. This fact requires specific control when using the breath acetone analyzer to measure breath acetone concentration to ensure that is only from diabetic disease; (2) Although diabetic patients, particularly T1D, have elevated mean breath acetone concentration in general, an individual breath acetone measurement is not sufficient for diabetes screening because of the high false positive rate that is partially due to the situations in (1). Our statistic analysis from the data collected from 22 T1D patients under the condition of fasting shows that the screening accuracy based on an individual breath acetone measurement using the breath analyzer is about 74%.

Nevertheless, this breath acetone analyzer still holds significant values in the following situations. (1) For T1D human subjects under a particular control, e.g., fasting, convenient and noninvasive breath acetone measurements can provide diabetes pre-screening with about 74% accuracy or better. This is also particularly valued for babies or children who have been diagnosed as T1D; breath acetone measurements as a complementary means can significantly reduce pain, cost, and inconvenience when using a BGM many times a day is required; (2) For diabetic patients with ketone bodies, breath acetone is a reliable indicator of the physiological symptoms and breath acetone level is strongly correlated to the blood β-hydroxybutyrade (BHB) level [41]; this breath analyzer can be a useful instrument for status monitoring; (3) For human subjects under intensive exercise, the analyzer can conveniently measure pre- and post-exercise breath acetone to evaluate the effect of exercise on burning fatty acids.

## 4. Summary

A GC-MS validated breath acetone instrument based on the CRDS technique has been used for breath acetone measurements in 386 human subjects. More than 1200 breath samples collected from 334 diabetic patients (22 T1D subjects and 312 T2D subjects) in a clinic and 52 non-diabetic healthy subjects in the research lab under different situations (fasting or post-meals) were measured by the validated breath acetone analyzer, LaserBreath-001; and blood glucose levels were simultaneously tested. The mean value of breath acetone concentration for the diabetic subjects (T1D and T2D) under fasting, 2 h post-breakfast, 2 h post-lunch, or 2 h post-dinner is all higher than that in the non-diabetic healthy subjects correspondingly and so is the total mean values. This result indicates that both T1D and T2D subjects have elevated mean breath concentrations. There was a linear correction (R = 0.56, *p* < 0.005) between the mean breath acetone concentration and the mean BG levels for 20 T1D subjects. No correlation was found between an individual breath acetone concentration and an individual BG level in T1D subjects, T2D subjects, and healthy subjects in this work.

The quantitative information about breath acetone levels was obtained by using a relatively large number of diabetic subjects involved in this work, which benefited from using the ringdown breath acetone analyzer with advantages of fast response, on-line measurement, and relatively low operation cost over the conventional GC-MS method. These features enable further clinical testing that may require a larger number of diabetic patients under various situations. The clinical application of breath acetone in pre-screening diabetes depends on improvement of the positive rate, which is about 74% currently for T1D. For diabetes with ketone bodies, the breath acetone concentration is a reliable and complimentary means in monitoring the blood BHB level. In this update, some data and findings are from our previous studies, some are from the most recent and unreported progresses, such as the latest version of the breath acetone analyzer, LaserBreath-001. The performance of the instrument on the measurement reproducibility (>95%) was improved by using a moisture filter in the breath sample instruction inlet. Further development efforts will be focused on more clinical testing; developments of algorithms to reduce interferences from other physiological parameters and of optimal conditions for specifications of measured breath acetone from diabetes only; and reduction of the instrument size, weight, and cost toward a POC device that can be used as a noninvasive means complementary to BG measurements in diabetes screening and management.

## Figures and Tables

**Table 1:** Breath samples collected from the 386 human subjects.

	Subject Number	T1D (*n* = 22)	T2D (*n* = 312)	Healthy (*n* = 52)
Sample Number Per Subject				
1		2	57	9
2		2	4	4
3		2	56	5
4		16	195	34
Total number of samples		76	1013	168

**Table 2:** Statistical information and test results of the 386 human subjects.

Factor		T1D (*n* = 22)	T2D (*n* = 312)	Healthy (*n* = 52)	*p* Value
Age		28.1 ± 13.4 (13.0~61.0)	57.0 ± 12.9 (14.0~83.0)	27.0 ± 4.8 (20.0~48.0)	<0.0001
Gender	male	8 (34.8%)	178 (57.1%)	22 (42.3%)	0.931
	female	14 (65.2%)	134 (42.9%)	30 (57.7%)	
Years (year)		5.5 ± 7.4 (0.1~23.0)	11.7 ± 8.0 (0.0~36.0)	—	—
Weight (kg)		54.8 ± 6.3 (46.0~69.0)	72.8 ± 14.0 (44.0~117.0)	62.2 ± 11.4 (46.0~91.0)	<0.0001
Height (cm)		163.1 ± 5.4 (156.0~171.0)	165.3 ± 8.6 (144.0~186.0)	167.5 ± 8.0 (153.0~183.0)	0.1
BMI (kg/m^2^)		20.6 ± 2.5 (18.4~27.2)	26.5 ± 3.8 (17.9~38.2)	22.1 ± 3.1 (17.1~33.9)	<0.0001
Acetone (ppm)	Fasting (n1 = 22, n2 = 312, n3 = 52)	6.9 ± 21.7 (0.7~103.7)	1.7 ± 0.7 (0.1~19.8)	1.3 ± 0.3 (0.3~1.9)	0.3
	2h post-breakfast (n1 = 20, n2 = 255, n3 = 40)	6.3 ± 19.6 (0.7~89.5)	1.5 ± 1.1 (0.1~7.1)	0.9 ± 0.5 (0.1~2.0)	<0.05
	2h post-lunch (n1 = 16, n2 = 195, n3 = 41)	4.0 ± 10.3 (0.3~42.5)	1.4 ± 1.0 (0.1~7.2)	1.0 ± 0.6 (0.1~2.0)	0.4
	2h post-dinner (n1 = 18, n2 = 251, n3 = 35)	1.5 ± 0.7 (0.6~2.9)	1.5 ± 1.3 (0.1~10.6)	1.1 ± 0.4 (0.3~1.4)	0.2
	Total (n1 = 76, n2 = 1013, n3 = 168)	4.9 ± 16 (0.3~103.7)	1.5 ± 1.3 (0.1~19.8)	1.1 ± 0.5 (0.1~2.0)	<0.01
BGL (mg/dL)	Fasting (n1 = 22, n2 = 312, n3 = 38)	203.3 ± 53.9 (86.4~324.0)	152.1 ± 41.0 (63.0~304.2)	91.8 ± 7.3 (79.2~108.0)	<0.0001
	2h post-breakfast (n1 = 20, n2 = 255, n3 = 35)	252.9 ± 70.4 (124.2~433.8)	206.5 ± 59.8 (73.8~392.4)	96.8 ± 13.6 (48.6~124.2)	<0.0001
	2h post-lunch (n1 = 16, n2 = 195, n3 = 32)	207.9 ± 70.4 (106.2~324.0)	189.0 ± 57.2 (73.8~405.0)	105.3 ± 14.0 (79.2~131.4)	<0.0001
	2h post-dinner (n1 = 18, n2 = 251, n3 = 34)	235.6 ± 93.3 (93.6~394.2)	181.4 ± 52.3 (90.0~397.8)	118.3 ± 12.4 (100.8~138.6)	<0.0001
	Total (n1 = 76, n2 = 1013, n3 = 168)	224.6 ± 74.7 (86.4~433.8)	180.6 ± 56.2 (63.0~405.0)	100.0 ± 14.5 (48.6~138.6)	<0.0001

NOTE Values presented as mean ± SD (min~max) with analysis of Kruskal-Wallistest or *n* (%) with Pearson chi-square test.
